# Anticipating & assessing adverse consequences of public health interventions - CONSEQUENT framework: Jan Stratil

**DOI:** 10.1093/eurpub/ckac130.218

**Published:** 2022-10-25

**Authors:** J Stratil, RL Biallas, A Movsisyan, K Oliver, EA Rehfuess

**Affiliations:** 1 LMU Munich, IBE, Munich, Germany; 2 Faculty of Public Health and Policy, LSHTM, London, UK

## Abstract

**Introduction:**

Despite the best intentions, public health (PH) interventions can have adverse and other unintended consequences (AUCs). AUCs may arise in novel PH interventions, as well as from known and tested PH interventions implemented in a new context. Despite their importance, this topic has been largely overlooked. Therefore, we used a structured value-guided as well as evidence-based approach, to develop a framework to support researchers, practitioners, and policy-makers in anticipating and assessing AUCs of PH interventions.

**Methods:**

We employed the ‘best-fit’ synthesis approach starting with an a priori framework and iteratively revising this based on systematically identified evidence. The a priori framework was derived from both the WHO-INTEGRATE framework and the Behaviour Change Wheel, to root the framework in global health norms and values, established mechanisms of PH interventions, and a complexity perspective. The a priori framework was advanced based on theoretical and conceptual publications and systematic reviews on the topic of AUCs in PH. Thematic analysis was used to revise the framework and identify new themes. To validate the framework, it was coded against four selected systematic reviews of AUCs of PH interventions.

**Results:**

The CONSEQUENT framework includes two components: the first focuses on AUCs and serves to categorise them; the second component highlights the mechanisms through which AUCs may arise. The first component comprises eight domains of consequences - health-related, health system, human and fundamental rights, acceptability- and adherence-related, equality- and equity-related, social and institutional, economic and resource-related, and environmental.

**Conclusions:**

Both over- and underestimation of AUCs of PH intervention poses risks. The CONSEQUENT framework may facilitate classification and conceptualization of AUCs of PH interventions during their development or evaluation to support evidence-informed decision-making.

**Key messages:**

- Despite their importance for evidence-informed decision making adverse and unintended consequences of public health interventions are often overlooked within public health research and practice.
- The CONSEQUENT framework can support decision-makers in anticipating, assessing, and accounting for adverse and other unintended consquences, as well as the machanisms leading to them.

